# Influence of *TPH2* and *HTR1A* polymorphisms on lifelong premature ejaculation risk among the chinese Han population

**DOI:** 10.1186/s12894-023-01222-9

**Published:** 2023-05-09

**Authors:** Fei Wang, Defan Luo, Jianxiang Chen, Cuiqing Pan, Zhongyao Wang, Housheng Fu, Jianbing Xu, Meng Yang, Shaowei Mo, Liying Zhuang, Weifu Wang

**Affiliations:** 1 Department of Urology, Hainan General Hospital, Affiliated Hainan Hospital of Hainan Medical University, No.19, Xiuhua Road, Xiuying District, Haikou, Hainan Province 570311 China; 2 Department of Lung Transplatation, The Second Affiliated Hospital of Hainan Medical University, Haikou, Hainan 571199 China; 3 Department of Urology, Affiliated Hospital of Xiangnan University, Chenzhou, Hunan 423000 China; 4 Ministry of Science and Education, Hainan Women and Children’s Medical Center, Haikou, Hainan 571100 China; 5 Library, Hainan Medical University, Haikou, Hainan 571199 China; 6 Department of Kidney Transplatation, The Second Affiliated Hospital of Hainan Medical University, Haikou, Hainan Province 571199 China

**Keywords:** Lifelong premature ejaculation (LPE), Case-control study, Single nucleotide polymorphism (SNP), *TPH2*, *HTR1A*, 5-hydroxytryptamine (5-HT)

## Abstract

**Background:**

Lifelong premature ejaculation (LPE) is one of the most common ejaculatory dysfunctions in men. The serotonin (5-HT) synthesis rate-limiting enzyme (*TPH2*) and receptor (*HTR1A*) in the 5-HT regulatory system may play a key role in the pathogenesis of LPE. However, there are few studies on the effects of *TPH2* and *HTR1A* polymorphisms on LPE risk. We speculated that *TPH2* and *HTR1A* polymorphisms may affect the occurrence and development of LPE in the Chinese Han population.

**Methods:**

In this study, 91 patients with LPE and 362 normal controls aged 18 to 64 years were enrolled in the male urology department of Hainan General Hospital in China from January 2016 to December 2018. The SNPs in *HTR1A* and *TPH2*, which are related to 5-HT regulation, were selected as indexes to genotype the collected blood samples of participants. Logistic regression was used to analyze the correlation between SNPs of *HTR1A* and *TPH2* with LPE susceptibility, as well as the relationship with leptin, 5-HT and folic acid levels.

**Results:**

The results revealed that *HTR1A*-rs6295 increased LPE risk in recessive model. Rs11178996 in *TPH2* significantly reduced susceptibility to LPE in allelic (odds ratio (OR) = 0.68, 95% confidence interval (95% CI) = 0.49–0.96, *p* = 0.027), codominant (OR = 0.58, 95% CI = 0.35–0.98, *p* = 0.040), dominant (OR = 0.58, 95% CI = 0.36–0.92, *p* = 0.020), and additive (OR = 0.71, 95% CI = 0.52–0.98, *p* = 0.039) models. G_rs11179041_T_rs10879352_ could reduce the risk of LPE (OR = 0.44, 95% CI = 0.22–0.90, *p* = 0.024) by haplotype analysis.

**Conclusion:**

*HTR1A*-rs6295 and *TPH2*-rs11178996 are associated with LPE risk in the Chinese Han population based on the finding of this study.

## Introduction

Premature ejaculation (PE) is one of the most common ejaculatory dysfunctions in males, and approximately 20 − 30% of males have experienced PE. PE seriously affects not only the physical and mental health of patients but also sexual partners, marital relations, and family stability [1, 2]. The onset of PE is not age-specific, and PE may occur in a large range of people from 18 to 64 years. PE can be divided into two types according to the nature and the time of onset, that is lifelong premature ejaculation (LPE) and acquired premature ejaculation (APE) [3, 4]. On the basis of the last international society for sexual medicine (ISSM) for the definition of PE: [1] ejaculation that always or nearly always occurs prior to or within about 1 min of vaginal penetration from the first sexual experience (LPE) or a clinically significant and bothersome reduction in latency time, often to about 3 min or less (APE); [2] the inability to delay ejaculation on all or nearly all vaginal penetrations; and [3] negative personal consequences, such as distress, bother, frustration, and/or the avoidance of sexual intimacy. [3]. It has been claimed that the prevalence rate of LPE is 5% globally and 3% in China [5]. Current PE treatment strategies include behavioral therapy, selective serotonin reuptake inhibitors and selective phosphodiesterase inhibitors [6]. Among them, drug therapy is the first-line treatment for PE [7]. Classical selective serotonin reuptake inhibitors (SSRIs), such as dapoxetine, citalopram and Fortacin™, effectively delay ejaculation in patients with lifelong PE [8–10]. Most PE treatments are either experimental or used off-label [6]. Therefore, finding its effective biomarkers is a new approach to treat PE.

Previous twin and familial studies have demonstrated a genetic susceptibility to LPE, with genetic factors accounting for 30% of twins [11, 12]. However, the genetic variations in which genes affect LPE susceptibility have not been elucidated [13]. Among them, the research of 5-hydroxytryptamine (5-HT) and its related regulatory genes has a very sufficient theoretical basis [14]. 5-HT is the most important neurotransmitter and has been found to regulate ejaculation [15]. Tryptophan hydroxylase (TPH) is an important enzyme for 5-HT synthesis, and its expression level directly influences the synthesis amount of 5-HT, which in turn affects the function of 5-HT [16]. As one of the subtypes of TPH, *TPH2* is specifically expressed in the raphe nucleus of 5-HT neurons, and regulates central 5-HT synthesis [17]. Studies have found that *TPH2* SNV019 and rs4290270 are significantly associated with LPE in the Han population [18]. Besides, 5-HT needs to bind to its receptors to exert its biological effects. Among these receptors, *HTR1A* (5-hydroxytryptamine receptor 1 A) is one of the 5-HT receptors and plays an indispensable role in the regulation of ejaculation [19]. Another study has confirmed that the objective diagnostic indicators of LPE, including leptin and folic acid, can participate in the regulation of the 5-HT regulatory system by affecting the metabolism of 5-HT, and the number and function of receptors and transporters [20, 21].

In general, 5-HT concentration and abnormal receptors are important causes of PE. Therefore, we speculated that the 5-HT synthesis rate-limiting enzyme (*TPH2*) and *HTR1A* may contribute to the development of PE. So far, there is a suggestive genome-wide association study (GWAS) of the association between 33 gene polymorphisms and LPE risk in Chinese Han males [22]. Moreover, there are many polymorphisms of *TPH2* and *HTR1A* genes, and most of them have been reported to be related to the occurrence of neurological diseases [23]. However, the effects of *TPH2* and *HTR1A* gene polymorphisms on LPE are rarely studied in the Chinese Han population. Hence, we further explored the mechanism by which *TPH2* and *HTR1A* gene polymorphisms affect LPE susceptibility in the Chinese Han population based on a case-control study. In addition, statistical analyses of LPE risk based on the levels of leptin, 5-HT, and folic acid were performed to identify potential risk factors for LPE. Our findings will provide a theoretical basis for further understanding of the pathogenesis of LPE.

## Materials and methods

### Study design

In this study, 91 patients with LPE and 362 normal controls aged 18 to 64 years were enrolled in the male urology department of Hainan General Hospital in China from January 2016 to December 2018. The SNPs in *HTR1A* and *TPH2*, which are related to 5-HT regulation, were selected as indexes to genotype the collected blood samples of participants. Logistic regression was used to analyze the correlation between SNPs of *HTR1A* and *TPH2* and EP susceptibility, as well as the relationship with leptin, 5-HT and folic acid levels.

### Ethical approval

This study was approved by the Ethics Committee of Hainan General Hospital and was conducted strictly in accordance with the World Medical Association Declaration of Helsinki. All participants signed an informed consent form after fully understanding the research purpose and protocol.

### Study participants

All participants were Han Chinese males with permanent sexual partners. The age, leptin, 5-HT and folic acid levels, premature ejaculation diagnostic tool (PEDT), intravaginal ejaculatory latency time (IELT) and international erectile function scale (IIEF-5) scores of the participants were analyzed. The case group met the following criteria: (1) PE time definition is less than 1 min for LPE, and less than 3 min for secondary PE; (2) the above symptoms that last longer than 6 months [24]. The control group met the following conditions: 1) the lasts more than 3 min for LPE from the first sexual life. The case and control groups excluded those who met the following criteria: (1) men with APE; (2) men with anatomical deformities of the genitals that severely impair sexual function; (3) other abnormalities of sexual function (such as erectile dysfunction); (4) men with severe psychological disorders which cannot be well controlled by treatment; (5) men with other diseases, such as diabetes, stroke, myocardial infarction, cardiovascular disease, cancer, etc. At the same time, LPE patients were regularly reviewed and followed up.

### SNPs selection and genotyping

Based on the 1,000 Genomes Project (http://www.1000genomes.org/) and dbSNP (https://www.ncbi.nlm.nih.gov/SNP/) database, three single nucleotide polymorphisms (SNPs) (rs878567, rs6294, and rs6295) in *HTR1A* and ten SNPs (rs11178996, rs11178997, rs11179001, rs10879346, rs1386492, rs11179023, rs7305115, rs11179041, rs10879352, and rs120074175) in *TPH2* were selected. The minor allele frequencies (MAFs) of these candidate SNPs were greater than 5% in the global population.

Genomic DNA was extracted from peripheral blood samples of study participants by a Gold Mag-Mini Whole Blood Genomic DNA Purification kit (Gold Mag Co. Ltd., Xi’an, China) in strict accordance with the instructions [25]. NanoDrop 2000 (Thermo Scientific, Waltham, Massachusetts, USA) was utilized to determine whether the concentration and purity of the extracted DNA meet the standards, and which can be used for further experiments [26]. The genotyping processes were performed on the Agena MassARRAY RS1000 (Shanghai, China) platform, and the SNP genotyping data were processed and analyzed by Agena Typer 4.0 software (version 4.0, Agena Bioscience, San Diego, CA, USA).

### Statistical analyses

The experimental data were statistically analyzed by Microsoft Excel, SPSS 20.0 (SPSS, Chicago, IL), and PLINK software (version 1.07) (http://pngu.mgh.harvard.edu/purcell/plink/). The Chi-square test was used to evaluate whether the distribution of polymorphisms in the control group meets Hardy-Weinberg equilibrium (HWE). Odds ratios (ORs) and 95% confidence intervals (95% CIs) were calculated to assess the correlation between SNPs and the risk of LPE based on logistic regression models. Linkage disequilibrium (LD) analysis and haplotype construction were performed by the Haploview software package (version 4.2) (https://www.broadinstitute.org/haploview/haploview), and the correlation between haplotypes and LPE susceptibility was examined using PLINK software [25].

## Results

### Characteristics of participants

In this study, the baseline data of 453 participants (91 LPE patients and 362 healthy people) including age and some clinical indexes were collected (Table 1). Age, PEDT, IELT, IIEF-5 score and 5-HT level were statistically significant between cases and controls (*p* < 0.001), and leptin and folic acid expression was not obvious different between cases and controls. The basic information of candidate SNPs included in this study is presented in Table 2. Except for *TPH2*-rs11179001 (*p* < 0.001), the other candidate SNPs in controls were in accordance with HWE (*p* > 0.01). Rs11178996 in *TPH2* was related to a lower risk of LPE in the allelic model. Individuals carrying the “G” allele had a reduced risk of LPE (OR = 0.68, 95% CI = 0.49–0.96, *p* = 0.027) compared with those carrying the “A” allele.

**Table 1:** Comparison of basic characteristics between cases and healthy controls

Variables	Control (%)	Case (%)	*p*-value
Total	362(79.91%)	91(20.09%)	
PEDT	3.17 ± 1.82	17.98 ± 2.79	< 0.001
IELT (s)	704.85 ± 339.07	70.73 ± 33.58	< 0.001
Age	41.28 ± 10.95	32.40 ± 6.99	< 0.001
IIEF-5 score	23.26 ± 1.33	22.27 ± 3.61	< 0.001
5-HT (ng/mL)	92.11 ± 97.71	39.47 ± 45.22	< 0.001
Leptin (ng/mL)	1.89 ± 1.40	1.71 ± 1.89	0.434
Folic acid (ng/mL)	60.96 ± 57.05	55.38 ± 46.91	0.519

PEDT, Premature ejaculation diagnostic tool; IELT, Intravaginal ejaculatory latency time; IIEF-5: international erectile function scale; 5-HT: 5-hydroxytryptamine

*p* values were calculated from t test

*p* < 0.05 indicates statistical significance

**Table 2:** *HTR1A* and *TPH2* candidate SNPs and association with risk of LPE in allele model

SNP	Chr	Position	Gene(s)	Role	Alleles	Frequency (MAF)		*p - HWE*	OR (95% CI)	*p* value
						Cases	Controls			
rs878567	5	63,960,164	*HTR1A*	ncRNA_intronic	G/A	0.181	0.184	0.034	0.98(0.65–1.50)	0.941
rs6294	5	63,961,426	*HTR1A*	exonic	T/C	0.181	0.184	0.034	0.98(0.65–1.50)	0.941
rs6295	5	63,962,738	*HTR1A*	ncRNA_intronic	G/C	0.203	0.193	0.011	1.07(0.71–1.61)	0.743
rs11178996	12	71,937,074	*TPH2*	intergenic	G/A	0.341	0.431	0.041	0.68(0.49–0.96)	**0.027** ^*^
rs11178997	12	71,938,373	*TPH2*	upstream	A/T	0.231	0.215	0.212	1.09(0.74–1.61)	0.655
rs11179001	12	71,944,865	*TPH2*	intronic	A/G	0.456	0.436	**<0.001**	1.08(0.78–1.50)	0.634
rs10879346	12	71,958,055	*TPH2*	intronic	T/C	0.407	0.429	0.335	0.91(0.65–1.27)	0.579
rs1386492	12	71,968,485	*TPH2*	intronic	T/C	0.434	0.367	0.572	1.32(0.95–1.84)	0.098
rs11179023	12	71,978,617	*TPH2*	intronic	A/G	0.17	0.200	0.324	0.82(0.53–1.26)	0.361
rs7305115	12	71,979,082	*TPH2*	exonic	A/G	0.44	0.384	0.657	1.26(0.91–1.75)	0.170
rs11179041	12	72,010,169	*TPH2*	intronic	A/G	0.198	0.802	0.216	0.89(0.60–1.34)	0.590
rs10879352	12	72,013,178	*TPH2*	intronic	C/T	0.198	0.802	0.218	0.88(0.59–1.32)	0.548
rs120074175	12	72,031,544	*TPH2*	exonic	A/G	0.192	0.808	0.210	0.90(0.59–1.35)	0.599

SNP: Single nucleotide polymorphism; HWE: Hardy-Weinberg equilibrium; ORs: Odds ratio; 95% CI: 95% confidence intervals;

^*^p - HWE obtained from Fisher’s exact test

### Relationship between *TPH2* and *HTR1A* polymorphisms and LPE risk

The associations of different genotypes of *TPH2* and *HTR1A* with LPE risk in multiple genetic models are shown in Table 3. The results showed that rs6295 in *HTR1A* was significantly associated with an increased risk of LPE in recessive model (“G/G” genotype: OR = 3.44, 95% CI = 1.03–11.54, *p* = 0.045) after adjustment. On the contrary, rs11178996 reduced the risk of LPE in codominant (“G/G” genotype: OR = 0.58, 95% CI = 0.35–0.98, *p* = 0.040), dominant (“A/G-G/G” genotype: OR = 0.58, 95% CI = 0.36–0.92, *p* = 0.020), and additive (OR = 0.71, 95% CI = 0.52–0.98, *p* = 0.039) models after adjustment.

**Table 3:** Logistic regression analysis of the association between *HTR1A* and *TPH2* gene polymorphisms and risk of LPE.

SNP	Model	Genotype	Control	Case	OR (95% CI)	*p*-value
rs6295	Co-dominant	C/C	228(63.2%)	59(64.8%)	1.00	
		C/G	127(35.2%)	27(29.7%)	3.22(0.95–10.92)	0.060
		G/G	6(1.7%)	5(5.5%)	0.82(0.50–1.36)	0.445
	Dominant	C/C	228(63.2%)	59(64.8%)	1.00	
		C/G-G/G	133(36.9%)	32(35.2%)	0.93(0.58–1.50)	0.767
	Recessive	C/C-C/G	355(98.4%)	86(94.5%)	1.00	
		G/G	6(1.7%)	5(5.5%)	3.44(1.03–11.54)	**0.045** ^*^
	Log-additive	---	---	---	1.08(0.70–1.65)	0.731
rs11178996	Codominant	A/A	127(35.1%)	44(48.4%)	1.00	
		A/G	158(43.6%)	32(35.2%)	0.56(0.29–1.08)	0.083
		G/G	77(21.2%)	15(16.5%)	0.58(0.35–0.98)	**0.040** ^*^
	Dominant	A/A	127(35.1%)	44(48.4%)	1.00	
		A/G-G/G	235(64.8%)	47(51.7%)	0.58(0.36–0.92)	**0.020** ^*^
	Recessive	A/A-A/G	285(78.7%)	76(83.6%)	1.00	
		G/G	77(21.2%)	15(16.5%)	0.73(0.40–1.34)	0.312
	Log-additive	---	---	---	0.71(0.52–0.98)	**0.039** ^*^

SNP: Single nucleotide polymorphism; ORs: Odds ratio; 95% CI: 95% confidence intervals;

^*^ Bold values indicate statistical significance (*p* < 0.05)

### Relationship between *TPH2* gene polymorphisms and LPE-related indicators

The relationship between *TPH2* gene polymorphisms and the levels of leptin, 5-HT, and folic acid in LPE patients was also analyzed (Table 4). Rs11178997 in *TPH2* was found to be correlated with leptin levels in codominant (*p* = 0.027) and dominant (*p* = 0.007) models. Rs10879346 in *TPH2* showed a significant association with folic acid levels in the recessive model (*p* = 0.037). Rs1386492 in *TPH2* showed a correlation with 5-HT levels in both codominant (*p* = 0.013) and recessive (*p* = 0.004) models. Rs11178996 in *TPH2* was related to the levels of leptin (*p* = 0.031) and folic acid (*p* = 0.032) in the dominant model.

**Table 4:** Relationship between candidate gene polymorphism and leptin, 5-HT, and folic acid levels in LPE patients

SNP	Model	Genotype	Leptin (ng/mL)	5-HT (ng/mL)	Folic acid
rs11178997	Co-dominant	TT(n = 29)	2.29 ± 2.21	40.06 ± 50.25	40.06 ± 50.25
		AT(n = 12)	0.75 ± 0.41	40.30 ± 43.24	40.30 ± 43.24
		AA(n = 7)	0.95 ± 0.82	35.61 ± 28.10	35.61 ± 28.10
		*p*-value	**0.027** ^*^	0.972	0.548
	Dominant	TT(n = 29)	2.29 ± 2.21	40.06 ± 50.25	52.91 ± 44.92
		AT-AA(n = 19)	0.83 ± 0.58	38.58 ± 37.57	59.16 ± 50.83
		*p*-value	**0.007** ^*^	0.913	0.657
	Recessive	TT-AT(n = 28)	0.95 ± 0.82	35.61 ± 28.10	73.50 ± 57.53
		AA(n = 7)	1.84 ± 1.99	40.13 ± 47.77	52.29 ± 44.98
		*p*-value	0.255	0.810	0.274
rs10879346	Co-dominant	TT(n = 16)	1.25 ± 1.29	54.26 ± 57.45	45.20 ± 40.79
		TC(n = 25)	1.97 ± 2.26	27.35 ± 27.16	52.40 ± 40.62
		CC(n = 7)	1.85 ± 1.52	48.96 ± 59.33	89.33 ± 69.47
		*p*-value	0.488	0.149	0.103
	Dominant	TT(n = 16)	1.25 ± 1.29	54.26 ± 57.45	45.20 ± 40.79
		TC-CC(n = 32)	1.94 ± 2.10	32.08 ± 36.53	60.47 ± 49.52
		*p*-value	0.232	0.110	0.293
	Recessive	TT-TC(n = 41)	1.85 ± 1.52	48.96 ± 59.33	89.33 ± 69.47
		CC(n = 7)	1.69 ± 1.96	37.85 ± 43.09	49.59 ± 40.33
		*p*-value	0.834	0.554	**0.037** ^*^
rs1386492	Co-dominant	CC(n = 17)	1.78 ± 2.20	35.40 ± 41.60	68.93 ± 56.25
		TC(n = 22)	1.79 ± 1.79	27.01 ± 29.24	51.42 ± 44.64
		TT(n = 9)	1.40 ± 1.64	77.63 ± 64.89	39.49 ± 25.63
		*p*-value	0.864	**0.013** ^*^	0.277
	Dominant	CC(n = 17)	1.78 ± 2.20	35.40 ± 41.60	68.93 ± 56.25
		T/C-TT(n = 31)	1.67 ± 1.73	41.71 ± 47.61	47.95 ± 40.00
		*p*-value	0.858	0.649	0.140
	Recessive	T/C-C/C(n = 39)	1.78 ± 1.95	30.67 ± 34.91	59.05 ± 50.11
		TT(n = 9)	1.40 ± 1.64	77.63 ± 64.89	39.49 ± 25.63
		*p*-value	0.587	**0.004** ^*^	0.264
rs11178996	Co-dominant	AA(n = 27)	1.96 ± 1.44	43.30 ± 57.21	83.64 ± 66.30
		AG(n = 13)	2.62 ± 2.89	33.86 ± 34.20	64.40 ± 49.40
		GG(n = 8)	1.20 ± 1.14	41.04 ± 47.47	42.67 ± 34.86
		*p*-value	0.073	0.87	0.065
	Dominant	AA(n = 27)	1.20 ± 1.14	41.04 ± 47.47	42.67 ± 34.86
		AG-GG(n = 21)	2.37 ± 2.42	37.46 ± 43.24	71.73 ± 55.63
		*p*-value	**0.031** ^*^	0.789	**0.032** ^*^
	Recessive	AA-AG(n = 40)	1.96 ± 1.44	43.30 ± 57.21	83.64 ± 66.30
		GG(n = 8)	1.66 ± 1.97	38.71 ± 43.29	49.73 ± 40.83
		*p*-value	0.687	0.796	0.061

SNP: Single nucleotide polymorphism; ORs: Odds ratio; 95% CI: 95% confidence intervals;

*p*-value calculated by logistic regression analysis with adjustments for gender and age;

^*^ Bold values indicate statistical significance (*p* < 0.05)

### Relationship between haplotypes and LPE risk

**Fig. 1:**
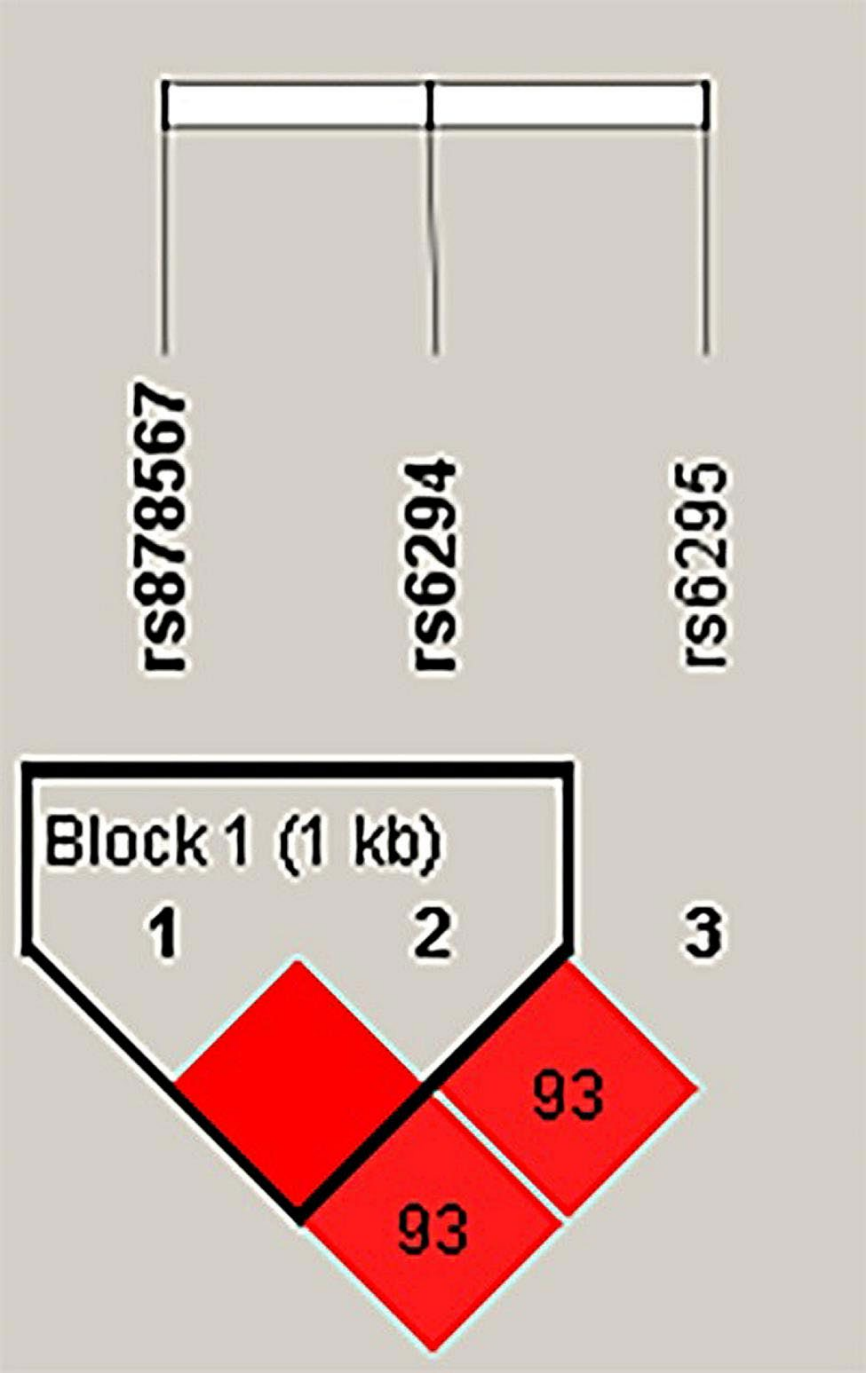
Haplotype block map of *HTR1A* SNPs. Block 1 includes rs878567 and rs6294 with D’ = 1 for the corresponding variants

Additionally, LD association analysis and haplotype construction of polymorphisms in *HTR1A* and *TPH2* were also carried out. As shown in Fig. 1, rs878567 and rs6294 in *HTR1A* have a close chain relationship and constitute a haplotype block.

**Fig. 2:**
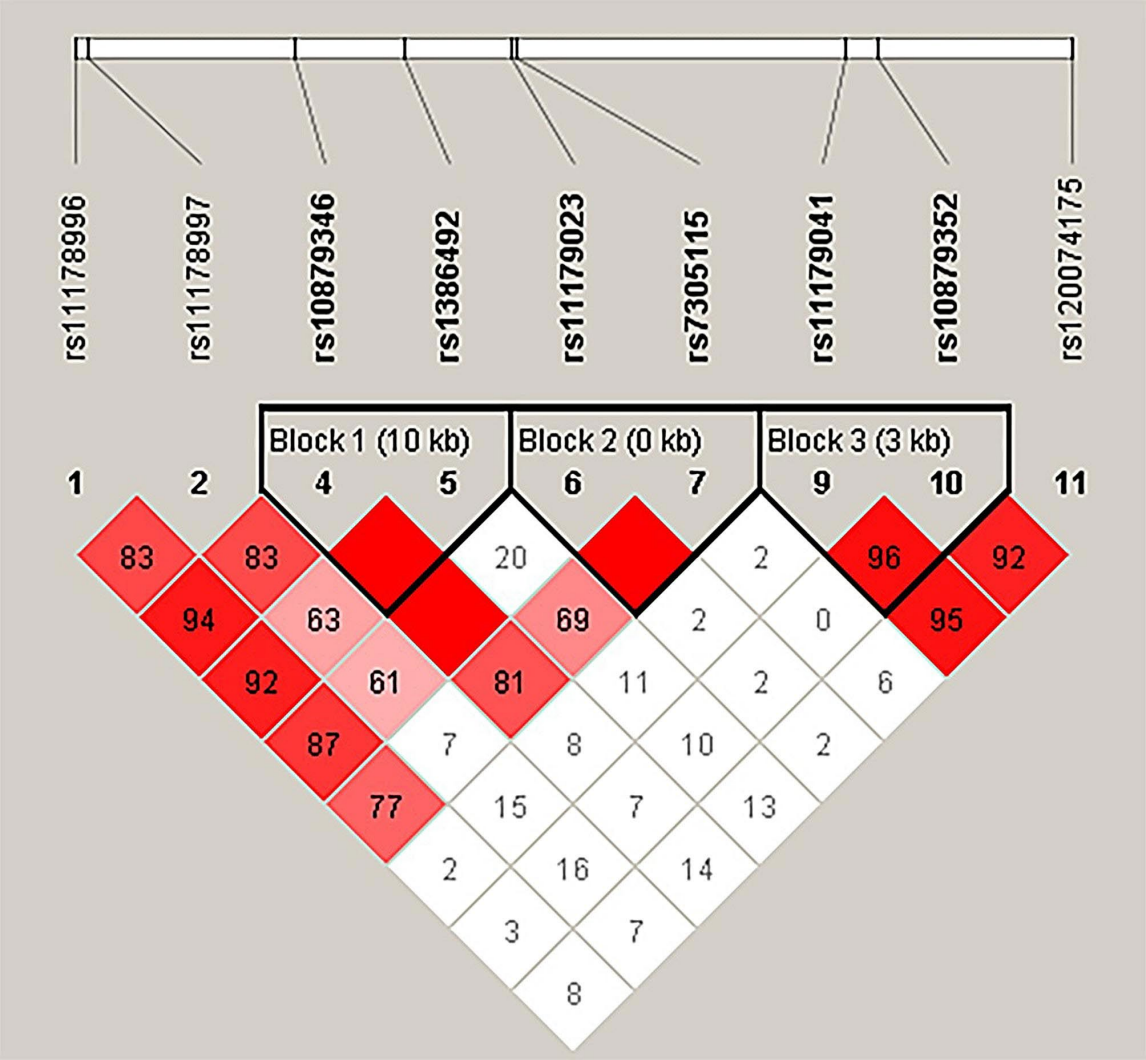
Haplotype block map of *TPH2* SNPs. Block 1 includes rs10879346 and rs1386492 with D’ = 1 for the corresponding variants. Block 2 includes rs11179023 and rs7305115 with D’ = 1 for the corresponding variants. Block 3 includes rs11179041 and rs10879352 with D’ = 1 for the corresponding variants

As shown in Fig. 2, rs10879346-rs1386492, rs11179023-rs7305115 and rs11179041-rs10879352 in *TPH2* have the close chain relationship and constitute three haplotype blocks.

Moreover, the haplotype *TPH2* G_rs11179041_T_rs10879352_ significantly reduced the risk of LPE after adjustment (OR = 0.44, 95% CI = 0.22–0.90, *p* = 0.024) (Table 5). The other haplotypes showed no significant correlation with LPE risk.

**Table 5:** *HTR1A* and *TPH2* haplotype frequencies and the association with LPE risk

Gene	SNPs	Haplotype	Freq (case)	Freq (control)	OR (95% CI)	*p*-value
*HTR1A*	rs878567|rs6294	GA	0.181	0.184	0.98(0.63–1.52)	0.938
		AT	0.181	0.184	0.98(0.63–1.52)	0.938
*TPH2*	rs10879346|rs1386492	AT	0.434	0.367	1.33(0.95–1.86)	0.093
		GC	0.407	0.429	0.91(0.66–1.27)	0.585
		AC	0.159	0.204	0.74(0.48–1.15)	0.179
	rs11179023|rs7305115	GT	0.440	0.384	1.28(0.91–1.79)	0.160
		AC	0.170	0.200	0.81(0.53–1.26)	0.351
		GC	0.390	0.416	0.90(0.65–1.25)	0.533
	rs11179041|rs10879352	AT	0.198	0.209	0.93(0.62–1.40)	0.737
		CC	0.198	0.224	0.86(0.58–1.28)	0.451
		GT	0.049	0.106	0.44(0.22–0.90)	**0.024** ^*^

Block comprised of the three closely linked SNPs rs9303628 and rs2054847; ORs: odds ratio, 95% CI: 95% confidence intervals;

^*^ Bold values indicate statistical significance (*p* < 0.05)

## Discussion

Studies have shown that genetic factors play an important role in LPE [11]. In our previous study, 13 genes (LACTBL1, SSBP3 and ACOT11) were found to be significantly associated with LPE risk in Chinese male Han population by genome-wide association analysis [22]. It was also reported that the genetic variation of 5-HT1B rs11568817 and 5-HT2C rs518147 were significantly associated with the occurrence of PE [27]. In this study, we investigated whether *TPH2* and *HTR1A* gene polymorphisms in the 5-HT regulatory system are potentially associated with LPE susceptibility. Our results further confirmed that rs6295 in *HTR1A* was associated with an increased risk of LPE, while rs11178996 in *TPH2* was related to a reduced risk of LPE. Different genotypes of rs11178997, rs10879346, and rs1386492 in the *TPH2* gene were significantly correlated with the levels of leptin, folic acid, and 5-HT, respectively. Different genotypes of rs11178996 in the *TPH2* gene were related to the levels of leptin and folic acid. The haplotype G_rs11179041_T_rs10879352_ showed an association with a decreased risk of LPE. This study preliminarily evaluated the effects of *TPH2* and *HTR1A* gene polymorphisms on the susceptibility to LPE in the Chinese Han population.

The *TPH2* gene is located on chromosome 12q21.1 and includes 11 exons [28]. The protein encoded by *TPH2* catalyzes the first and rate-limiting steps in the biosynthesis of serotonin which is an important hormone and neurotransmitter. Currently, *TPH2* gene polymorphisms have been extensively studied as potential predisposing factors for mental illnesses such as major affective disorder [29, 30], and major depressive disorder [28, 31]. Studies have reported that rs11178997, rs10879346, rs11179023, rs7305115, and rs120074175 in the *TPH2* gene are all closely related to the occurrence of depression [31, 32]. The allele A of rs11178997 affects the expression of *TPH2* by inhibiting its transcriptional activity in neurons, resulting in reduced 5-HT synthesis and depression [33]. The functional polymorphism rs120074175, in which arginine at position 441 in the coding region of the human gene is replaced by histidine, can cause 80% loss of *TPH2* function, which in turn reduces 5-HT synthesis and triggers depression [34]. It is remarkable that our study explored the correlation between *TPH2* polymorphisms and the risk of LPE. Though we did not find any significant correlation between rs11178997, rs10879346, rs11179023, rs7305115, and rs120074175 and the risk of LPE, we discovered that different genotypes of rs11178997, rs10879346, and rs1386492 were significantly related to the levels of leptin, folic acid, and 5-HT, respectively. Moreover, different genotypes of rs11178996 were associated with the levels of leptin and folic acid. Most importantly, there has been no research reported on rs11178996. Our study demonstrated that rs11178996 was correlated with decreased risk of LPE.

The *HTR1A* gene is located on chromosome 5q11.2-q13, encodes G protein-coupled receptors for 5-HT (serotonin), and belongs to the 5-HT receptor subfamily [35, 36]. The main function of *HTR1A* is to regulate the release of serotonin and the metabolism of dopamine and serotonin. It has been reported that *HTR1A* affects the occurrence of several diseases including periodic fever, menstrual cycle-dependence febrile episode [37], generalized anxiety disorder [38], and schizophrenia [39]. Previous studies have suggested that *HTR1A* polymorphisms are associated with various mental diseases, such as major depressive disorder [40], obsessive-compulsive disorder [41], and anxiety disorder [42]. There are few studies on the relationship between *HTR1A* polymorphisms and LPE. Until 2014, Janssen et al. [23] have revealed for the first time that *HTR1A*-rs6295 is related to the length of IELT in patients with LPE in the Dutch Caucasian population. Recently, Roaiah et al. [43] have explored the potential relationship between *HTR1A*-rs6295 and LPE risk in Egyptians, and have found that the genotype of patients with LPE is mostly “CG”, while the genotype of the controls is “GG”. Our study is the first to investigate the impact of *HTR1A* rs878567, rs6294, and rs6295 on the risk of LPE in the Chinese Han population. Our results indicated that the genotype of patients with LPE was mostly “CC”, which is consistent with the study reported by Janssen et al. [23] in the Dutch population, while is contrary to the study by Mohamed Farid Roaiah et al. [43] in the Egyptian population. This may be caused by genetic differences among people of different races. Although we did not discover any association of rs878567 and rs6294 with the risk of LPE, we noticed that rs6295 was correlated with an increased risk of LPE. Individuals carrying the “GG” genotype had a 3.44-fold increased risk of LPE compared with those carrying the “C/C-C/G” genotype. Rs6295 is located in the promoter region of the 5-*HT1A* gene, and can regulate the region-specific modification of *HTR1A* expression and transcription. Some researchers have claimed that the rs6295-G allele can bind to nuclear transcription factors, enhance the expression of 5-*HT1A* autoreceptors, and reduce the release of 5-HT to affect the risk of LPE [44, 45]. Therefore, we speculated that the genotype “GG” of rs6295 may also influence 5-HT metabolism by regulating the expression level of *HTR1A*, and thus it was related to the risk of LPE.

In this study, we explored the correlation between *HTR1A* and *TPH2* gene polymorphisms and LPE susceptibility in Chinese Han males, and found that *HTR1a*-rs6295 increased the LPE risk in recessive models, while *TPH2*-rs11178996 was a protective factor for LPE occurrence. In addition, *TPH2* SNPs were associated with leptin, 5-HT, and folic acid levels, and haplotype Grs11179041Trs10879352 showed a reduced risk of LPE. This study further demonstrated that genetic variation plays an important role in the occurrence and development of LPE, which is related to the expression of leptin, 5-HT and folic acid. Furthermore, it provides the basis and guidance for the research on the mechanism of genes and genotypes regulating the occurrence, development and treatment of LPE. Simultaneously, it also excavates new biomarkers and provides theoretical support for the clinical diagnosis, prevention and personalized treatment of LPE.

Our current study also has some limitations due to the small sample size, limited information on the clinical indicators of LPE, and the single study population which is only the Chinese Han population. Therefore, it is necessary to expand the sample size and carefully design high-quality studies to further validate our findings. At the same time, the anthropometric variables, nutritional status, duration of sexual relationships, total physical activity, smoking, alcohol consumption, drinking, psychological status and any drugs history of the participants in this study were missing not available for statistical analysis, and confounding factors should be further excluded in later studies. In addition, our samples only represent the Chinese Han population. Due to the genetic differences among different ethnic groups, we will collect more samples from various populations to verify the correlation between *TPH2* and *HTR1A* gene polymorphisms and LPE risk.

## Conclusion

To sum up, our study provided powerful evidence that *HTR1A*-rs6295 and *TPH2-*rs11178996 were significantly associated with the risk of LPE, and the haplotype G_rs11179041_T_rs10879352_ shows a decreased risk of LPE in the Chinese Han population. Studies have suggested that *TPH2* and *HTR1A* polymorphisms may play a potential role in the development of LPE, which provides data support for the prevention, diagnosis and personalized treatment of LPE.

## Data Availability

The datasets generated and/or analysed during the current study are available in the zenodo repository (https://www.zenodo.org/record/7726589#.ZA6W-flDSUk).

## Declarations

### Human accordance

All experimental protocols followed the Declaration of Helsinki.

### Study approval

The study was approved by the Ethics Committee of Hainan General Hospital.
